# Immunological Identification and Characterization of the Capsid Scaffold Protein Encoded by UL26.5 of Herpes Simplex Virus Type 2

**DOI:** 10.3389/fcimb.2021.649722

**Published:** 2021-05-26

**Authors:** Xueqi Li, Jianbin Wang, Tangwei Mou, Yang Gao, Lichun Wang, Shengtao Fan, Xingli Xu, Guorun Jiang, Pingfang Cui, Xiangxiong Xu, Suqin Duan, Jingjing Zhang, Dandan Li, Yun Liao, Li Yu, Heng Zhao, Ming Lu, Hailian Zhu, Ran Gu, Ying Zhang, Wei Dong, Qihan Li

**Affiliations:** ^1^ Institute of Medical Biology, Chinese Academy of Medicine Sciences & Peking Union Medical College, Yunnan Key Laboratory of Vaccine Research and Development for Severe Infectious Diseases, Kunming, China; ^2^ Reproductive & Gynecology Department, The First People’s Hospital of Yunnan Province, Kunming, China

**Keywords:** herpes simplex virus type 2, ICP35, 2-D protein electrophoresis, immunization, viral replication

## Abstract

Herpes simplex virus type 2 (HSV2), a pathogen that causes genital herpes lesions, interferes with the host immune system *via* various known and unknown mechanisms. This virus has been used to study viral antigenic composition. Convalescent serum from HSV2-infected patients was used to identify viral antigens *via* 2-D protein electrophoresis and immunoblotting. The serum predominantly recognized several capsid scaffold proteins encoded by gene UL26.5, mainly ICP35. This protein has been primarily reported to function temporarily in viral assembly but is not expressed in mature virus particles. Further immunological studies suggested that this protein elicits specific antibody and cytotoxic T lymphocyte (CTL) responses in mice, but these responses do not result in a clinical protective effect in response to HSV2 challenge. The data suggested that immunodominance of ICP35 might be used to design an integrated antigen with other viral glycoproteins.

## Introduction

Herpes simplex virus type 2 (HSV2), a human alpha-herpesviridae virus (Shukla and Spear, 2001), is known to spread in teenage and adult populations and to cause herpetic pathogenic lesions in the skin of the genitals (Looker et al., 2015), which can seriously impact quality of life (Castillo et al., 2020). Epidemic data have recently suggested that more than 400 million people are infected with HSV2 worldwide (James et al., 2020). Approximately 45 million infected patients have been identified in the United States, and this number increases by 1 million patients each year (Mathew and Sapra, 2020). Given this background and the lack of specific treatment methods (Sandgren et al., 2020), the development of HSV2 vaccines has become a major concern for the control of this disease (Spicknall et al., 2019); however, no applicable vaccine for HSV2 has been reported to date, although some clinical trials of a HSV2 vaccine have been initiated (Corey et al., 1999; Belshe et al., 2012). Based on the data from these trials, no protective effect has been observed in the individuals immunized with these experimental vaccines, although neutralizing antibodies were detected in serum samples (Corey et al., 1999; Belshe et al., 2012). These results suggest that our knowledge about how the HSV2 antigen trains the immune system to recognize and target the invading virus is lacking. Studies of HSV2 pathogenesis have indicated that various proteins expressed during viral infection are capable of interacting with the host immune system and interfering with antiviral immunity (Suazo et al., 2015), although these viral proteins should be recognized by the immune system and elicit specific responses, such as specific antibody responses against each viral protein (Tognarelli et al., 2019). Thus, understanding the mechanism of host immunity against this virus depends upon integrated analyses of the immune recognition of the majority of its proteins and the roles played by these proteins during infection. Based on this hypothesis, antibodies were used in neutralizing assays and enzyme-linked immunosorbent assays (ELISAs) to evaluate the immunological reactions that occur during HSV2 infection; however, these methods were insufficient to show the relationships of various viral proteins and immune serum. Here, we used protein 2-D electrophoresis and immunoblotting associated with immunofluorescent assay to investigate the recognition of viral proteins by convalescent serum collected from HSV2-infected patients. Interestingly, we found that several viral capsid scaffold proteins encoded by genes UL26.5 and UL26, especially ICP35, were predominantly recognized by the convalescent serum compared with other viral structural proteins that elicit neutralizing antibodies (Cairns et al., 2015). Moreover, further immunological characterization of ICP35 suggested the possibility that immunodominance might be used to design an integrated antigen with other viral glycoproteins.

## Materials and Methods

### Virus and Cells

The HSV2 HG52 strain was purchased and preserved by the Virus Immunization Room of the Institute of Medical Biology, Chinese Academy of Medical Sciences. The virus was grown in Vero cells (ATCC, Manassas, USA), which were cultured in an incubator at a constant temperature of 37°C using Dulbecco’s modified Eagle medium (DMEM; Corning, NY, USA) supplemented with 5% fetal bovine serum (FCS; HyClone, Logan, USA), 100 U/ml penicillin, and 100 µg/ml streptomycin.

### Viral Titration

All experimental procedures were performed under BSL-2 laboratory conditions. Virus samples were serially diluted 10-fold with serum-free DMEM. Different viral dilutions were added to a 96-well plate. Each dilution (100 µl per well) was added to 8 parallel wells. Then, 100 µl of Vero cell suspension was added to each well at a concentration of 2.5 × 10^5^ cells/ml. After the plate was incubated at 37°C in 5% CO_2_ for 7 days, the cytopathic effect (CPE) was observed and assessed with an inverted microscope (Nikon, Tokyo, Japan).

### Neutralizing Antibody Test

Heat-inactivated serum was diluted and coincubated with live virus (100 lgCCID_50_/well) for 2 h at 37°C, and then, 100 μl Vero cells (10^5^/mL) were added. Then, the plates were incubated at 37°C in 5% CO_2_ for 7 days. The CPEs were observed and assessed with an inverted microscope (Nikon) to determine the neutralizing antibody titer of the serum. The geometric mean titers (GMTs) of the neutralizing antibodies were measured.

### Plaque Assay

HSV2 virus (MOI=1) and serum samples were added to a 6-well plate with a confluent monolayer of cells and coincubated for 2 h. Then, the plate was incubated at 37°C in 5% CO_2_ for 2-3 days after a mixture of 2× DMED and 2% methylcellulose was added. Plaques could be observed after staining with crystal violet dye.

### Viral Purification

Monolayers of Vero cells were infected with HSV2 at a MOI of 1, and at 48 h postinfection, the virus liquid was harvested. The virus liquid was concentrated by Millipore Centricon Plus-70. The concentrated virus liquid was layered on a 15 to 55% iodixanol gradient and centrifuged at 30,000 rpm in an SW41 rotor for 2.5h. The purified virus samples were observed *via* electron microscopy and were harvested at -80°C.

### Immunofluorescence

When the 293 cells were 70%-90% confluent, they were transfected with the eukaryotic vectors of intact ICP35, VP22a and VP24 according to the Lipofectamine™ 3000 Reagent Protocol in glass bottom cell culture dishes, and the samples were harvested at 18h. In addition, Vero cells were infected with the HSV2 virus at an MOI of 0.2, and the samples were harvested at 4h, 12h, 24h postinfection. These dishes were fixed in 4% paraformaldehyde for 30 min and then blocked using 4% bovine serum albumin (BSA). For detection of the HSV-2 ICP35, VP22a, VP24 antigen and trace ICP35 protein during viral infection, the dishes were sequentially incubated with primary antibody convalescent serum, immune serum of ICP35 and secondary antibody Alexa Fluor^®^ 647 Goat pAb to Rb IgG (Lot: GR33281 42-4, Abcam), Alexa Fluor^®^ 488 Goat anti-human IgG (H+L) (Lot: 2196582, Invitrogen). All cell nuclei were detected with DAPI. Fluorescence was visualized and analyzed using a confocal microscope (TCS SP2, Leica).

### Co-Immunoprecipitation

Vero cells were infected with the HSV2 virus at an MOI of 1. At the indicated time points (8, 14 and 20 h p.i.), the cells were washed with PBS and lysed in 1 ml RIPA lysis buffer for 10 min. The lysates were centrifuged (12,000 rpm, 4°C for 10 min) and precleared with 50 μl of control agarose beads. After centrifugation, the cell lysate supernatants were incubated overnight with the anti-ICP35 antibody or negative control at 4°C with rotation. Pretreated protein A + G beads (50 μl) were added, and the samples were incubated at 4°C for an additional 2 h and then washed five times and eluted with 500 μl of Tris-EDTA buffer containing 1% SDS. The DNA was then extracted and analyzed using real-time PCR. The specific primer sets used are listed in **Table 1**.

**Table 1:** The qRT-PCR primers and ICP35 plasmid construction.

Name	Sequence (5’ - 3’)
HSV2-Gg-F	CGCTCTCGTAAATGCTTCCCT
HSV2-Gg-R	TCTACCCACAACAGACCCACG
HSV2-Gg-Probe	FAM-CGCGGAGACATTCGAGTACCAGATCG-BHQ1
HSV-2-UL26.5-F	ATAGAATTCATGAACCCCGTTTCGGCATC
HSV-2-UL26.5-R	GCGAAGCTTTTTATTTAAAATATATGAAAACACACA

F, Forward primer; R, Reverse primer.

### 2D Protein Electrophoresis and Western Blot Analysis

Purified HSV2 virus samples were lysed with 2D protein lysis buffer (8 M urea, 4% CHAPS, 100 mM DTT, and 2% IPG buffer). The total protein concentration was determined using a bicinchoninic acid (BCA) protein concentration determination kit (Beyotime Biotechnology, Shanghai, China) according to the manufacturer’s instructions. Approximately 80 μg of protein in 170 µl of hydration solution was separated with the IPGphor instrument based on their pI values using immobilized linear gradient strips (Immobiline™ DryStrip, GE Healthcare, USA) covering the pH range 4-7. Then, the protein was separated by 12% sodium dodecyl sulfate polyacrylamide gel electrophoresis (SDS-PAGE). The proteins were transferred to polyvinylidene fluoride (PVDF) membranes, blocked with 5% skim milk (prepared in TBST), incubated with convalescent serum from clinically diagnosed HSV2 patients, and incubated with peroxidase-conjugated AffiniPure rabbit anti-human IgA + IgG + IgM (H+L) (Jackson, USA). The PVDF membranes were incubated with an ECL ultrasensitive chemiluminescence reagent and placed in a Bio-Rad gel imager for exposure and color development.

### Silver Stain

The reaction system of each glue in the silver dyeing process is 250ml. The dyeing procedure and the preparation of the solution required for each step are as follows: fix (25ml acetic acid, 100ml methanol, 125ml milli-Q water) twice, 15 minutes each time; sensitize (75ml methanol, 0.5g Na_2_S_2_O_3_, 17g NaAc, milli-Q water to 250ml) for 30 minutes; rinse (250ml milli-Q water) 3 times, 5 minutes each time; silver dyeing (0.625g AgNO_3_, milli-Q water to 250ml) 30 minutes; rinse (250ml milli-Q water) 2 times, 1 minute each time; color development (6.25g Na_2_CO_3_, 100μl formaldehyde, milli-Q water to 250ml) 4 minutes; stop solution (3.65g EDTA, milli-Q water to 250ml) for 10 minutes; after rinsing (250ml milli-Q water) for 1 minute, use Image Scanner III to scan and save the pictures, and use ImageMaster 2D platinum 7.0 software to analyze and screen out the required protein gel spots.

### ICP35 Recombinant Protein

The ICP35 recombinant protein was constructed with the prokaryotic expression vector pET-32a (+) (Solarbio, Beijing, China), which was purified with the Beyotime His Tag Protein Product Purification Kit (Beyotime Biotechnology, China). The sequences of the primers used are listed in **Table 1**.

### Animals

Mice: Four-week-old female BALB/c mice (Beijing Vital River Laboratory Animal Technologies Co. Ltd., Beijing, China) were used in this study. The mice were anesthetized by inhalation of 2% isoflurane for all the procedures, and every effort was made to minimize suffering. The mice were bred in strict accordance with the standard procedures for breeding laboratory animals.

### Ethics

Human experiments: Convalescent serum samples were collected from patients diagnosed with HSV2 infection at the First Affiliated Hospital of Kunming Medical University, and the patients provided informed consent. The protocols were reviewed and approved by the Experimental Management Association of the IMB, CAMS (approval number: DWSP 201803018-1).

Animal experiments: The animal experiments were designed and performed according to the principles in the ‘‘Guide for the Care and Use of Laboratory Animals” and in the ‘‘Guidance for Experimental Animal Welfare and Ethical Treatment”. The protocols were reviewed and approved by the Experimental Animal Management Association of the IMB, CAMS (approval number: DWSP 201902030-1). All the animals were completely under the care of veterinarians at the IMB, CAMS. The study was adhered to standard biosecurity and institutional safety procedures and has appropriated equipment and facilities.

### Immunization and Viral Challenge

Mice were randomly divided into three groups and intramuscularly immunized with 20 µg of purified ICP35 (the purified ICP35 protein concentration was determined by BCA) (n=30) or with Al (OH)_3_ adjuvant alone (n=30) used as a control, and PBS negative control (n=10), on days 0, 28 and 42. The serum samples were obtained at 7 and 28 days after immunization. The mice were infected with HSV2 virus on day 7 after the booster injection except negative control. HSV2 infection (2x10^4^ CCID_50_/mouse) *via* the vaginal route was performed on the mice immunized with the ICP35 protein or with the adjuvant control. All the animals were monitored daily to observe their clinical symptoms. For weight observation group (n=10 per group), the mice were monitored daily after challenge to observe body weight and survival rate. For experimental sampling group (n=20 per group), the routine vaginal secretion, tissue and blood samples were collected 1 day, 3 days, 5 days, 7 days and 15 days after infection for subsequent testing and neutralization assays. Three mice were euthanized in each group at one time point. All remaining mice were euthanized at the end of the experiment.

### ELISPOT

An ELISPOT assay was performed with the Mouse IFN-γ/IL-4 ELISPOT Kit (Mabtech, Cincinnati, OH, USA) according to the manufacturer’s protocol. Briefly, peripheral blood mononuclear cells (PBMCs) were isolated from mouse spleens by lymphocyte isolation (Dakewe, China) and plated in duplicate wells. Purified HSV2 ICP35 recombinant protein and the positive stimulus phytohemagglutinin (PHA) were added at a concentration of 5 µg/well to 96-well plates precoated with IFN-γ and IL-4. The plates were incubated at 37°C for 24 h in a carbon dioxide incubator. Then, the cells were removed, and the spots were developed. The colored spots were counted with an ELISPOT reader (CTL, Shaker Heights, OH, USA).

### Quantification of the Viral Load by q-RT-PCR

Total DNA was extracted from blood and tissue samples from the experimental mice with an Axygen^®^ AxyPrep Body Fluid Viral DNA/RNA Miniprep Kit (Axygen^®^, USA). According to the protocol, the primers used for q-RT-PCR were designed to be specific for the gG sequences in the HSV2 genome (**Table 1**). q-RT-PCR was performed using the Takara Premix Ex Taq^™^ (Probe qPCR) Kit (TaKaRa, China).

### Electron Microscopy

HSV2 virus was purified by iodixanol density gradient centrifugation. Postfixation, en bloc contrasting, dehydration and embedding in epoxy resin were done following a standard protocol (Laue, 2010). Samples were stained with 1% phosphotungstic acid and observed by transmission electron microscopy (Hitachi, Kyoto, Japan).

### Histopathological Examinations

The organs of the experimental mice were fixed in 10% formalin, embedded in paraffin, sliced into 4-μm-thick sections and stained with hematoxylin and eosin (H&E). The morphology was assessed with an inverted microscope (Nikon).

### Statistical Analysis

The data are shown as the mean and standard deviation. GraphPad Prism software (San Diego, CA, USA) and the *t test* were used for the statistical analyses.

## Results

### Recognition of Viral-Encoded Proteins by Convalescent Serum From HSV2-Infected Patients

**Figure 1:**
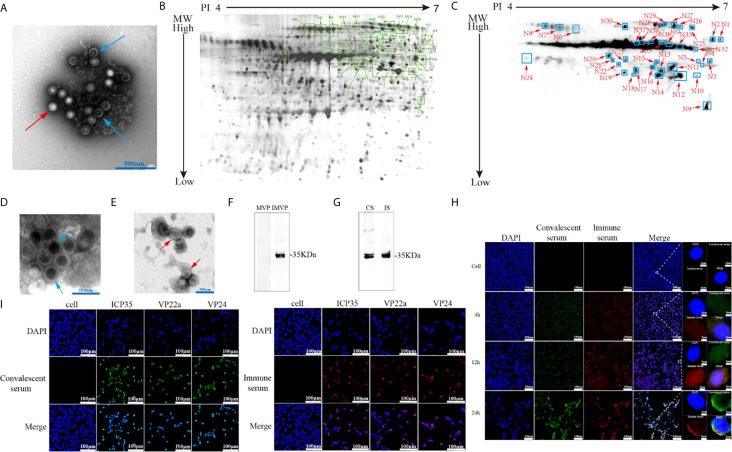
Viral-encoded proteins were recognized by the convalescent serum from HSV2-infected patients. **(A)** Electron microscopy of purified HSV2 virus; the blue arrow indicates immatured virus particles and the red arrow indicates matured virus particles. **(B)** 2-D electrophoresis of purified HSV2 virus. The glue was stained by silver dyeing. The number marked in the figure represents the protein neutralized by HSV2 patient convalescent serum according with **(C)**. The specific details are shown in the **Table 2**. MW refers to Molecular weight; PI refers to Isoelectric point. **(C)** Immunoblotting showed that the convalescent serum could identify viral proteins in the purified HSV2 virus. **(D)** Electron microscopy of purified HSV2 virus; the blue arrow indicates immatured virus particles. **(E)** Electron microscopy of purified HSV2 virus; the red arrow indicates matured virus particles. **(F)** The immune-blotting of matured and immatured virus particles with convalescent serum. The results showed that only trace levels of ICP35 were detected in matured particles compared to uncompleted particles. MVP refers to matured virus particles. IMVP refers to immatured virus particles. The convalescent serum was used as the primary antibody, and the secondary antibody was horseradish peroxidase labeled goat anti-human IgG (H+L) antibody. **(G)** The immune-blotting of the prokaryotic expressed ICP35 with convalescent serum and immune serum of ICP35. The convalescent serum and immune serum were used as the primary antibody, and the secondary antibody was horseradish peroxidase labeled goat anti-human IgG (H+L) antibody and goat anti-rabbit IgG (H+L) antibody. CS refers to convalescent serum. IS refers to immune serum of ICP35. **(H)** The results of the immunofluorescence detection with convalescent serum and immune serum of expressed ICP35 after HSV-2 virus infection. Vero cells were infected with HSV-2 at a MOI of 0.2, and at 4h, 12h, 24h postinfection, the samples were harvested. Blue represents nuclear staining (DAPI); green (Alexa Fluor^®^ 488 Goat anti-human IgG) represents staining with the convalescent serum; red (Alexa Fluor^®^ 647 Goat pAb to Rb IgG) represents staining with the immune serum. **(I)** The results of the immunofluorescence detection with convalescent serum and immune serum of ICP35 after transfecting eukaryotic vectors containing ICP35, VP22a and VP24 into 293 cells respectively. Blue represents nuclear staining (DAPI); red (Alexa Fluor^®^ 647 Goat pAb to Rb IgG) represents staining with the immune serum; green (Alexa Fluor^®^ 488 Goat anti-human IgG) represents staining with the convalescent serum.

Specific neutralizing antibodies, as certain indicators of antiviral immunity, reflect the recognition of key viral antigens, the main structural proteins that enable a virus to bind to receptors in the cell membrane, by the host immune system (Klasse, 2014). We performed protein 2-D electrophoresis and immunoblotting experiments with convalescent serum that was prepared by mixing 5 sera samples and showed a neutralizing antibody titer of 1:32 (**Supplemental Table 1**). Purified HSV2 was produced and harvested from infected Vero cells at different time points and was shown to contain various particles including those from different infectious stages (**Figure 1A**). This viral infectious sample was recognized by the convalescent serum, as indicated by the complicated antigenic map with multiple spots (**Figures 1B, C**). These spots were further identified using mass spectrometry, and various molecules of different sizes encoded by genes UL26.5 and UL26, including ICP35, VP22a and VP24, were recognized in the immunoblots (**Table 2**). In addition, other viral proteins, including some viral molecules that function during infection, such as TK, and structural components of the capsid, were recognized; however, only weak signals of glycoproteins that elicit neutralizing antibodies were observed in the blots (**Table 2**). Because ICP35 was thought to exist in immatured virus particles (Thomsen et al., 1995), we further purified the virus harvest using isodensity centrifugation to separate two different particles and performed immune-blotting with convalescent serum. The results showed that only trace levels of ICP35 were detected in matured particles compared to the other particles (**Figures 1D–F**), while the prokaryotic expressed ICP35 was recognized by convalescent serum in immune-blotting, either by immune serum of ICP35 (**Figure 1G**). To identify these data, we using convalescent serum and immune serum of expressed ICP35 traced the proteins encoded by UL26 gene during viral infection, the result of immune-fluorescent observation suggested that both sera were capable of recognizing the proteins encoded by UL26 gene including ICP35 during viral infection (**Figure 1H**). While the intact ICP35, VP22a and VP24 expressed in 293 cells were recognized by both sera (**Figure 1I**). These data comprehensively suggested that the viral proteins including ICP35 encoded by UL26 gene showed distinct immunogenicity during infection.

**Table 2:** Mass spectrometric identification of 2-D protein gel electrophoresis spots.

Spot number	Gene	Uniprot ID	Description
N1	MCP	P89442	Major capsid protein
N2	MCP	P89442	Major capsid protein
N3	UL26.5	G9I248	Capsid scaffolding protein
N4	UL42	P89463	DNA polymerase processivity factor
N5	UL26.5	G9I248	Capsid scaffolding protein
N6	TK	P89446	Thymidine kinase
N7	TK	P89446	Thymidine kinase
N8	TK	P89446	Thymidine kinase
N9	DBP	P89452	Major DNA-binding protein
N10	UL26.5	G9I248	Capsid scaffolding protein
N11	UL26.5	G9I248	Capsid scaffolding protein
N12	UL26.5	G9I248	Capsid scaffolding protein
N13	UL26.5	G9I248	Capsid scaffolding protein
N14	UL26.5	G9I248	Capsid scaffolding protein
N15	UL26.5	G9I248	Capsid scaffolding protein
N16	UL26.5	G9I248	Capsid scaffolding protein
N17	UL26.5	G9I248	Capsid scaffolding protein
N18	UL26.5	G9I248	Capsid scaffolding protein
N19	UL26.5	G9I248	Capsid scaffolding protein
N20	DBP	P89452	Major DNA-binding protein
N21	DBP	P89452	Major DNA-binding protein
N22	DBP	P89452	Major DNA-binding protein
N23	DBP	P89452	Major DNA-binding protein
N24	UL26.5	G9I248	Capsid scaffolding protein
N25	DBP	P89452	Major DNA-binding protein
N26	DBP	P89452	Major DNA-binding protein
N27	DBP	P89452	Major DNA-binding protein
N28	DBP	P89452	Major DNA-binding protein
N29	UL26.5	G9I248	Capsid scaffolding protein
N30	DBP	P89452	Major DNA-binding protein
N31	DBP	P89452	Major DNA-binding protein
N32	UL42	P89463	DNA polymerase processivity factor
N33	UL42	P89463	DNA polymerase processivity factor
N34	UL42	P89463	DNA polymerase processivity factor
N35	DBP	P89452	Major DNA-binding protein
N36	DBP	P89452	Major DNA-binding protein
N37	DBP	P89452	Major DNA-binding protein
N38	gD	Q69467	Envelope glycoprotein D

### ICP35 Elicits a Specific T Cell Response in HSV2-Infected Mice

**Figure 2:**
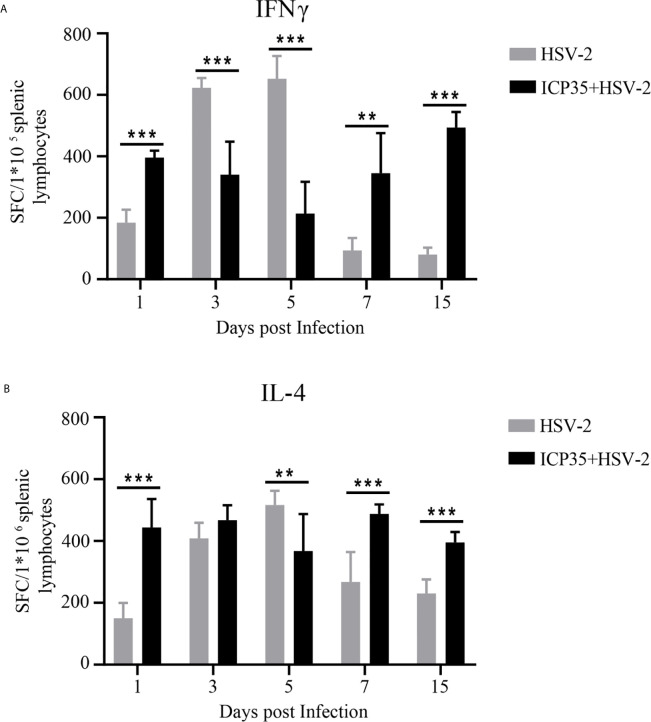
ICP35 elicited specific T cell response in mice. **(A)** The ELISPOT analysis of ICP35 elicited IFN-γ-secreting T cell responses within the splenic lymphocyte population in the immunized and control groups. Samples were run in duplicate. (n=3). **(B)** The ELISPOT analysis of ICP35 elicited IL-4- secreting T cell responses within the splenic lymphocyte population in the immunized and control groups. Samples were run in duplicate. (n=3). HSV-2, indicates the animal immunized with Al (OH)_3_ and challenged with HSV-2 groups; ICP35+HSV-2, indicates the animal immunized with ICP35 and challenged with HSV-2 groups;. SFC, indicates the Spot-forming Cells. **P<0.01, ***P<0.001.

Based on the ICP35 antigenic interaction with the antibodies in the convalescent serum, we used the prokaryotic expression method to produce a purified ICP35 protein and observed the relationship between its antigenicity and antiviral immunity in HSV2-infected mice. In ELISPOT assays to examine IFN-γ and IL-4 specificity, T cells isolated from the spleens of BALB/c mice infected with HSV2 were stimulated with the ICP35, and the proliferating T cell clones were counted. The results indicated that T cell stimulation with ICP35 protein clearly led to the proliferation of clones producing IFN-γ and clones producing IL-4. Similar results were obtained when T cells were stimulated with viral antigens (**Figures 2A, B**). This result suggested that the immune memory effect elicited by ICP35 played a role in host antiviral immunity after HSV2 infection.

### Study of the Immune Response Elicited by ICP35

**Figure 3:**
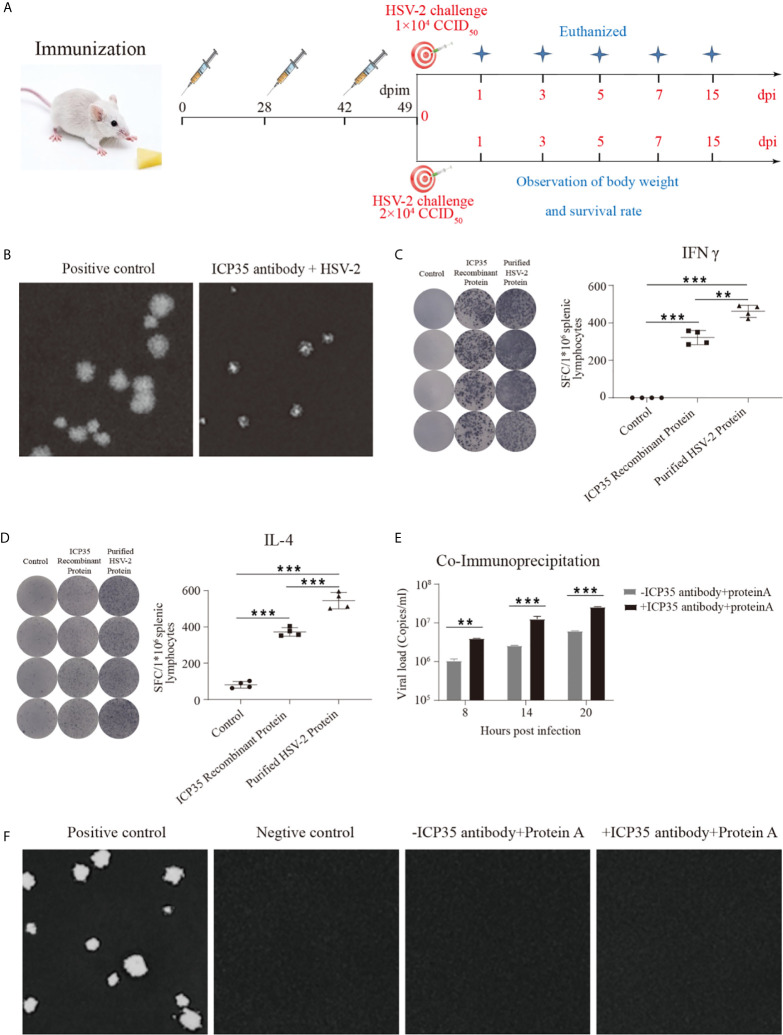
The integrated immune responses elicited by ICP35. **(A)** Schematic of ICP35 immunization and viral challenge protocols in the mice. **(B)** The addition of anti-ICP35 antibody restricted the sizes of the plaques. The positive control refers to HSV2 and normal serum samples; ICP35 antibody+HSV-2 refers to HSV2 and anti-ICP35 serum samples. **(C)** The ELISPOT analysis of ICP35 elicited IFN-γ-secreting T cell responses in ICP35 protein-immunized mice at day 28 after the last immunization (n=3). The spot diagram (left) and result (right) of IFN-γ were shown. Samples from the ICP35-immunized animals were stimulated with the ICP35 recombinant protein and purified HSV-2 protein. Samples were run in duplicate. **(D)** The ELISPOT analysis of ICP35 elicited IL-4-secreting T cell responses in ICP35 protein-immunized mice at day 28 after the last immunization (n=3). The spot diagram (left) and result (right) of IL-4 were shown. Samples from the ICP35-immunized animals were stimulated with the ICP35 recombinant protein and purified HSV-2 protein. Samples were run in duplicate. **(E)** Fluorescence quantitative PCR of protein A agarose gel particle sediment. -ICP35 antibody + protein A: the cell lysate supernatants infected HSV2 were incubated overnight without the anti-ICP35 antibody at 4°C; +ICP35 antibody + protein A: the cell lysate supernatants infected HSV2 were incubated overnight with the anti-ICP35 antibody at 4°C. **(F)** Plaque assay of protein A agarose gel particle sediment. The positive control refers to infection with HSV2 virus. The negative control refers to cells. -ICP35 antibody + protein A: the cell lysate supernatants infected HSV2 were incubated overnight without the anti-ICP35 antibody at 4°C; +ICP35 antibody + protein A: the cell lysate supernatants infected HSV2 were incubated overnight with the anti-ICP35 antibody at 4°C. **P<0.01, ***P<0.001.

To further immunologically characterize ICP35, we used BALB/c mice immunized with the prokaryotically expressed ICP35. The mice were administered three inoculations of 20 µg/dose into muscular tissue at intervals of 4 and 2-weeks (**Figure 3A**). The neutralizing antibodies were detected using a plaque assay at days 7 and 28 after the last immunization, and no positive results were observed at these two time points; however, the addition of serum to the assay restricted the sizes of the plaques, even if it did not decrease the virus titers (**Figure 3B**). ELISPOT was used to detect IFN-γ and IL-4 production at day 28 post immunization, and the results suggested a positive T cell response against this protein and against the purified virus harvested from infected Vero cells (**Figures 3C, D**). In a further study of the interaction between the serum and the viral antigen, viral particles harvested from infected Vero cells were incubated with the serum and were shown to contain viral genome but were noninfectious (**Figures 3E, F**). These results suggested that ICP35 is an effective viral antigenic protein but is not a neutralizing antigen.

### Immunization With ICP35 Attenuates the Clinical Symptoms in Mice Challenged With HSV2

**Figure 4:**
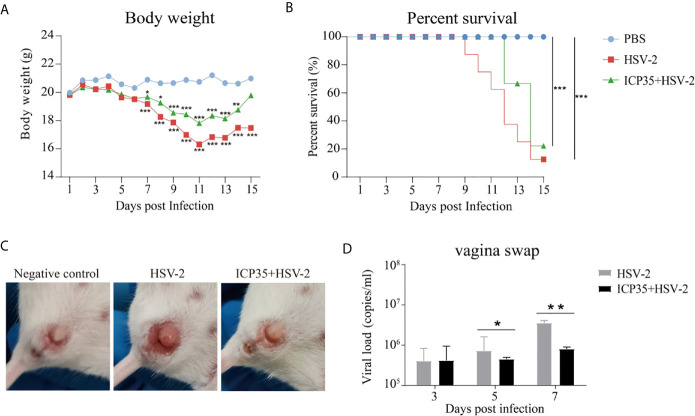
Immunization with ICP35 attenuated the clinical symptoms in mice challenged with HSV2. **(A)** The mice were monitored daily after challenge to observe their symptoms (n=10/group). Decreased body weight was observed in the immunized with ICP35 (ICP35+HSV-2), with Al (OH)_3_ adjuvant groups (HSV-2) and PBS negative control groups (PBS), but beginning on day 7, this trend differed between the experimental and control groups. The data are the mean of ten independent animals. *P<0.05, **P<0.01, ***P<0.001 vs. PBS. **(B)** The survival rate of mice challenged with HSV2 was monitored daily (n=10/group). Death occurred in the Al (OH)_3_ adjuvant groups (HSV-2) beginning on day 9, and death occurred in the ICP35 immunized group (ICP35+HSV-2) beginning on day 11. ***P<0.001. **(C)** The clinical symptoms of the mice challenged with HSV2 in the immunized with ICP35 (ICP35+HSV-2) and with Al (OH)_3_ adjuvant groups (HSV-2). PBS group without challenge as negative control. **(D)** The detection of the viral load by vaginal swab in the immunized with ICP35 (ICP35+HSV-2) and with Al (OH)_3_ adjuvant groups (HSV-2) after viral challenge. The data are the mean ± SD of three independent animals.

To immunologically characterize HSV2, we infected BALB/c mice immunized with the expressed ICP35 protein with HSV2. The HSV2 HG52 strain was used to infect two groups of immunized mice *via* the vaginal route. The low-dose group received 1 x 10^4^ CCID_50_/mouse while the high-dose group, which was used to assess the survival rate, received 2 x 10^4^ CCID_50_/mouse, and similar groups were established as positive controls (**Figure 3A**). After infection, the mice were monitored daily to observe their symptoms. The mice in the immunized and control groups presented decreasing body weights. Beginning on day 7, this trend differed between the experimental and control groups (**Figure 4A**). By day 15, the experimental and control groups showed similar survival rates (**Figure 4B**). Interestingly, a difference was observed in the local tissues of the infected vaginas. In the immunized mice, these tissues presented weaker inflammatory reactions without exudation, but in the control mice, these tissues presented obvious inflammation with exudation (**Figure 4C**). The detection of viral load *via* vaginal swabs also supported the clinical features described above (**Figure 4D**). These results suggested that the immune response elicited by ICP35 contributed, to some extent, to antiviral immunity.

### Viral Proliferation and the Resulting Pathological Damage in Various Tissues of ICP35 Protein-Immunized Mice

**Figure 5:**
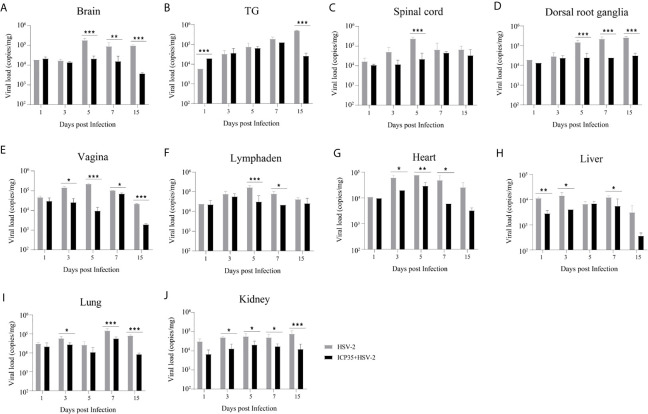
Viral proliferation in various tissues of the ICP35 protein-immunized or control mice. **(A–J)** The viral loads in various tissues of the immunized mice with ICP35 (ICP35+HSV-2), especially those in the vaginal tissues, were lower than those in various tissues of the mice with Al(OH)_3_ adjuvant (HSV-2). **(A)** Brain, **(B)** TG, **(C)** Spinal cord, **(D)** Dorsal root ganglion, **(E)** Vagina, **(F)** Lymphaden, **(G)** Heart, **(H)** Liver, **(I)** Lung, **(J)** Kidney. The data shown in **(A–J)** are the mean ± SD of three independent animals. *P<0.05, **P<0.01, ***P<0.001.

**Figure 6:**
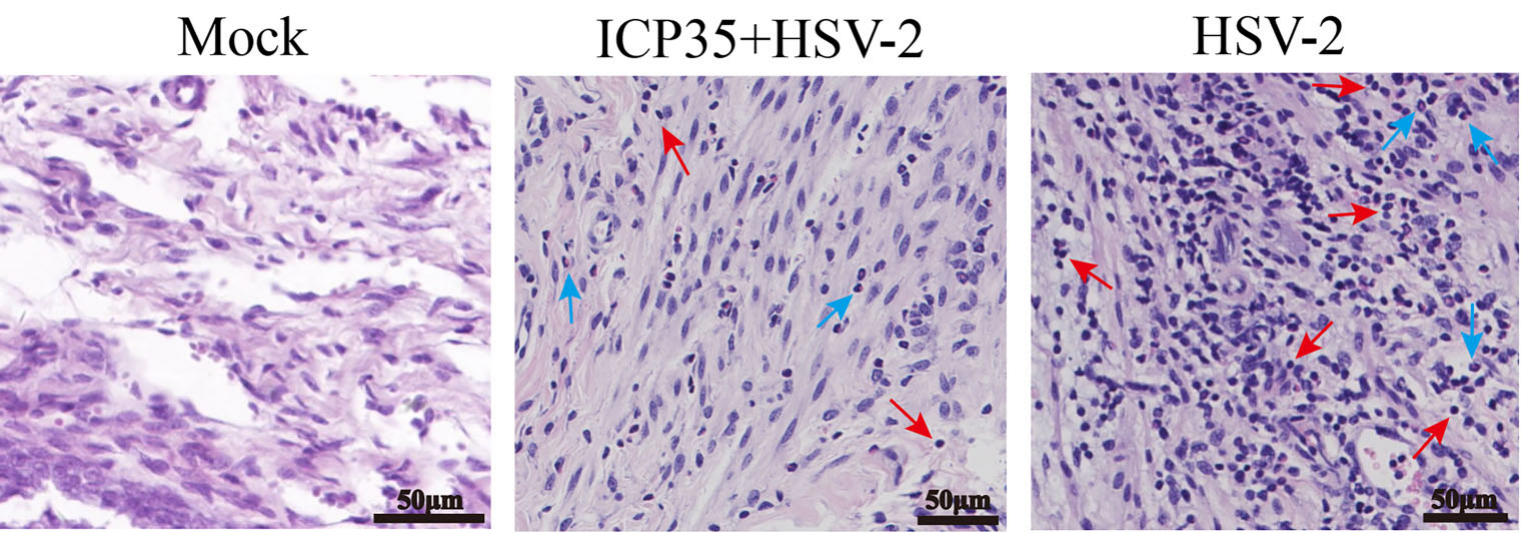
The resulting pathological damage in various tissues of the ICP35 protein-immunized or control mice. Histopathological observation of vaginal epithelial tissue from the PBS negative control (Mock), immunized with ICP35 (ICP35+HSV-2) and immunized with Al (OH)_3_ adjuvant mice (HSV-2) after viral challenge. Inflammatory cell infiltration (red arrow: lymphocytes, blue arrow: neutrophils).

We sacrificed the immunized and control mice at 1, 3, 5, 7 and 15 days after low-dose viral challenge and detected the viral loads of various tissues. The results suggested that the viral loads in various tissues of the immunized mice, especially that in the vaginal tissue, were lower than those in the positive control mice, although these particles showed similar dynamic proliferative processes (**Figures 5A–J**). Histopathological observation suggested that viral challenge led to varying degrees of pathological inflammatory reactions in various tissues in the immunized and control mice (**Table 3**). Observation of the local vaginal tissue did not reveal invasive pathological lesions in the epithelial structure of the immunized mice, although there was infiltration of inflammatory cells; however, invasive tissue lesions associated with inflammatory reactions were found in the vaginal epithelial tissues of the control mice (**Figure 6**). These results suggested that the immunity induced by ICP35 did restrict viral pathogenesis to some extent.

**Table 3:** Histopathological observation of pathological inflammatory reactions in various tissues in the immunized or control mice after viral challenge.

Group	Tissues	Histopathological observation
HSV-2	Brain	+
	TG	–
	Spinal cord	–
	Vagina	+++
	Lymphaden	+
ICP35 protein+HSV-2	Brain	+
	TG	–
	Spinal cord	–
	Vagina	++
	Lymphaden	+

-, Indicates no obvious abnormality; +, Indicates a small amount of inflammatory cell infiltration or bleeding; ++, Indicates medium inflammatory cell infiltration or bleeding, +++; Indicates lots of inflammatory cell infiltration or bleeding.

## Discussion

HSV2, a pathogen that spreads in populations *via* sexual contact and causes herpes progenitalis (Ahmed et al., 1982), has been studied due to its various strategies for interfering with the host immune system (Peng et al., 2009; Stefanidou et al., 2013; Stempel et al., 2019). Although our accumulated knowledge about these strategies allows us to understand some mechanisms by which the virus evades immune recognition and avoids immune attack, there are some unknown mechanisms that hinder the strategic study of HSV2 vaccines (Johnston et al., 2016). For instance, although specific neutralizing antibodies are elicited and are an important immunological indicator for the evaluation of individuals infected or immunized with HSV2 or vaccine antigens, these antibodies do not provide clinical protective effects during virus infection (Diebolder et al., 2014), suggesting a more complicated mechanism by which the virus interferes with the immune system. Based on this background, our work first investigated the recognition of virus-encoded protein antigens in infected cells by antibodies in the convalescent serum from HSV2-infected patients *via* 2-D electrophoresis and immunoblotting. We expected to primarily identify the remnant viral structural proteins with significant immunogenicity. Interestingly, we observed a clear trend that the antibodies in the convalescent serum recognized the viral capsid scaffold protein ICP35 and other similar proteins encoded by genes UL26.5 and UL26. ICP35 is thought to be a capsid structural protein that temporarily functions in capsid during viral assembly (Thomsen et al., 1995). Importantly, ICP35 actually is not expressed in mature virion (Thomsen et al., 1995), which means that it does not contribute to the antigenicity or immunogenicity of mature HSV2 virion. Our further work using the prokaryotic expression method and immunological studies characterized the antigenicity of this protein and showed that the elicited immunity was unable to control viral proliferation in mouse tissues *via* antibody and specific cytotoxic T lymphocyte (CTL) responses, although it did attenuate the clinical symptoms of local tissues to some extent. These data provided us with the technical possibility that the immunodominance of ICP35 might be used to design an integrated antigen with other viral glycoproteins, but further studies are needed to clarify the function of this protein in viral interference with host immunity.

## Data Availability Statement

The original contributions presented in the study are included in the article/**Supplementary Material**. Further inquiries can be directed to the corresponding authors.

## Ethics Statement

The studies involving human participants were reviewed and approved by Experimental Management Association of the IMB, CAMS. The patients/participants provided their written informed consent to participate in this study. The animal study was reviewed and approved by Experimental Animal Management Association of the IMB, CAMS.

## Author Contributions

QL, YZ, and WD conceived and designed the study. XL and JW performed the experiments and analyzed the data. TM, YG, YZ, LW, SF, XLX, GJ, PC, XXX, SD, JZ, DL, YL, LY, HZ, ML, HLZ, RG contributed reagents, materials and analysis tools. QL wrote and edited the manuscript. All authors contributed to the article and approved the submitted version.

## Funding

This work was supported by the National Natural Science Foundation of China (No. 31670173 and No.81802868) and grant from the Major Science and Technology Special Projects of Yunnan Province (No.202002AA100009).

## Conflict of Interest

The authors declare that the research was conducted in the absence of any commercial or financial relationships that could be construed as a potential conflict of interest.
