# Supplementary material for: Immunological Identification and Characterization of the Capsid Scaffold Protein Encoded by UL26.5 of Herpes Simplex Virus Type 2
Source: Front Cell Infect Microbiol. 2021 May 26;11:649722. doi: 10.3389/fcimb.2021.649722 (PMC8187855; doi:10.3389/fcimb.2021.649722)
Supplement: Supplementary file 1 [file Table_1.docx]

Supplementary Material

**Supplemental Table 1**

| **Name** | **Neutralizing antibody titers (GMTs)** |
| --- | --- |
| Convalescent serum samples 1 | 1:8 |
| Convalescent serum samples 2 | 1:32 |
| Convalescent serum samples 3 | 1:64 |
| Convalescent serum samples 4 | 1:16 |
| Convalescent serum samples 5 | 1:64 |
| Convalescent serum samples 1-5 (mix) | 1:32 |

**Supplementary Table 1.** The table indicates the neutralizing antibody titers of 5 convalescent serum samples. GMTs indicate the geometric mean titers.
